# Safety and efficacy of en bloc transurethral resection versus conventional transurethral resection for primary nonmuscle-invasive bladder cancer: a meta-analysis

**DOI:** 10.1186/s12957-019-1776-4

**Published:** 2020-01-04

**Authors:** Dong Zhang, Lifeng Yao, Sui Yu, Yue Cheng, Junhui Jiang, Qi Ma, Zejun Yan

**Affiliations:** 1 Department of Urology & Nephrology, Ningbo First Hospital, The affiliated Hospital of Zhejiang University, 59, Liuting Street, Ningbo, Zhejiang, China; 2 Department of Urology, Medical School of Ningbo University, Zhejiang, China

**Keywords:** Bladder tumor, En bloc transurethral resection, Conventional transurethral resection of bladder tumor, Meta-analysis, Complication

## Abstract

**Background:**

The purpose of this meta-analysis is to compare the safety and efficacy of en bloc transurethral resection of bladder tumor (EBRT) versus conventional transurethral resection of bladder tumor (CTURBT).

**Methods:**

We performed a meta-analysis of relevant articles through November 2019 using PubMed, Embase, and Cochrane Central Register to compare the safety and efficacy of EBRT versus CTURBT. The main endpoint included the operation time (OT), hospitalization time (HT), catheterization time (AT), perioperative period complications, bladder detrusor muscle found in the specimen, the residual tumor on the base, the ratio of the same site recurrence, and 12/24/36-month recurrence rate. Cochrane Collaboration’s Revman software, version 5.3, was used for statistical analysis.

**Results:**

A total of 19 studies with 2651 patients were included, 1369 underwent EBRT and 1282 underwent CTURBT. Patients treated with EBRT had a significantly lower AT, HT, obturator nerve reflex, bladder perforation, bladder irritation, postoperative complications, and 24-month recurrence rate than those who underwent CTURBT. While no significant difference was found in terms of OT, the ratio of bladder detrusor muscle found in the specimen, the residual tumor on the base, 12-month recurrence rate, 36-month recurrence rate, and the ratio of the same site recurrence. In mitomycin subgroup, EBRT was superior to CTURBT in terms of 12/24-month recurrence rate. Similarly, in the prospective subgroup and retrospective subgroup, EBRT had a lower 24-month recurrence rate than CTURBT. However, no significant difference was found in the low, intermediate, and high-risk group in the light of 12–36-month recurrence rate.

**Conclusions:**

Based on the included 19 articles, EBRT had a significantly lower AT, HT, intraoperative and postoperative complications, and 24-month recurrence rate than those treated with CTURBT. Well-designed randomized controlled trials were needed to reevaluate these outcomes.

**Trial registration:**

This meta-analysis was reported in agreement with the PRISMA statement and was registered on PROSPERO 2019 CRD42019121673.

## Background

Bladder cancer is one of the most common diseases in the genitourinary system. Approximately 54,9393 new bladder cancer patients have been diagnosed all over the world in 2018, with 19,9922 cases estimated cancer deaths. In other words, bladder tumor accounts for 7% of new cancer diagnoses and 4% of new estimated deaths in men [1, 2]. For nonmuscle-invasive bladder cancer (NMIBC), conventional transurethral resection of bladder tumor (CTURBT) combined with intravesical chemotherapy or Bacille Calmette-Guerin (BCG) is the standard treatment [3]. The identification of detrusor muscle in the specimen is an important factor for future treatment and prognosis. However, staging is often inaccurate because of charring of the resected tissues and absence of detrusor by TURBT. Complications such as obturator nerve reflex and bladder perforation may happen during the resection of lateral wall tumors, which is associated with urinary extravasation and neoplasm seeding [4]. Furthermore, the bladder tumor resected into fragments is contrary to the principle of tumor-free technique. Engilbertsson et al. had demonstrated that CTURBT induced the bladder tumor cell into the blood circulation [5]. However, it is now unknown whether CTURBT will increase the rate of metastatic disease. After the TURBT, the probability of recurrence rate reaches 15–61% in 1 year for TaT1, depending on the EORTC score and incomplete resection [3]. Thus, in order to achieve the complete resection, en bloc transurethral resection of bladder tumor (EBRT) has been gradually applied in the treatment of bladder tumor during the past years [3]. It has the ability to resect neoplasm with a 1 cm margin from the tumor base and precisely separate detrusor muscle as well as connective tissue and comply with oncological principles. In addition, the capacity to remove the neoplasm may yield the merit of shorter intervention time, because it avoids piece-by-piece removal by CTURBT, additionally prolonged by necessity to perform repeated hemostasis so as to improve visibility [6, 7].

HybridKnife, needle electrode, and laser are the main methods for EBRT. A series of studies comparing EBRT and CTURBT have been reported [6–24]. Although the clinical recurrence rate between two approaches is controversial, less intraoperative and postoperative complications have been observed when undergoing EBRT. A meta-analysis published in 2016 revealed that EBRT had a lower 24-month recurrence rate than CTURBT [25]. However, four recent randomized controlled trials (RCTs) revealed that no significant difference was found in terms of 12-month, 18-month, or 24-month recurrence rate [7, 12–14]. Therefore, an updated meta-analysis with robust evidence is needed. We aimed to synthesize the evidence-based data to assess the safety and efficacy of EBRT versus CTURBT for NMIBC.

## Methods

### Inclusion and exclusion criteria

The inclusion criteria were as follows: retrospective/prospective/RCT trails; English language; full-text articles; and studies that compared EBRT with CTURBT in the treatment of primary NMIBC (Ta,T1,Tis). The diagnosis of NMIBC was demonstrated by cystoscopy or histological evaluation of tumor tissue. Case-reports, reviews, abstracts, animal experiments, and letters were excluded.

### Literature search and data sources

We performed subject terms (MeSH) including “urinary bladder neoplasms” with their single words to search for relevant articles through November 2019 in PubMed, Embase, and Cochrane Central Register. The complete search used for PubMed was (urinary bladder neoplasms [MeSH terms] OR urinary bladder neoplasms [Text word]) AND (en bloc resection OR laser OR needle electrode OR endoscopic submucosal dissection OR Hybrid knife) AND (conventional transurethral resection of bladder tumor OR TURBT). The reference lists of relevant studies were also checked to identify potential records. Literature search and screening articles were achieved by two authors independently. The consensus was reached by discussion if there was any disagreement.

### Data extraction

One reviewer noted the study authors, date of publication, level of evidence, surgical method, tumor size, number of patients treated with EBRT or ETURBT, tumor grade, tumor T-stage, the method of intravesical instillation, operation time (OT), catheterization time (AT), hospitalization time (HT), obturator nerve reflex, bladder perforation, bladder irritation, bladder detrusor muscle, postoperative complications, residual tumor on base, 12/24/36-month recurrence rate, and same site recurrence rate. Dates were then verified by another reviewer.

### Quality assessment and statistical analysis

The Evidence-Based Medicine in Oxford was used to assess the level of evidence of all included articles [26]. Cochrane risk of bias tool was used to assess the methodological quality of included RCTs [27]. Furthermore, according to the Newcastle-Ottawa scale (including patient selection, comparability of the study groups, and assessment of outcome) [28], we could assess the methodological quality of non-randomized controlled trials.

Dichotomous or continuous data on OT, AT, HT, obturator nerve reflex, bladder perforation, bladder irritation, bladder detrusor muscle, postoperative complications, the residual tumor on base, 12/24/36-month recurrence rate, and same site recurrence were analyzed through Review Manager software, version 5.3 (Cochrane Collaboration, Oxford, United Kingdom). Subgroup analyses were performed based on study type and different therapy of intravesical instillations. Mantel-Haenszel chi-square test and *I*^2^ statistic were performed to assess the impact of study heterogeneity on the result of the meta-analysis. If the *P* value was > 0.1 and *I*^2^ < 50%, the fixed-effect model was performed. Otherwise, the random effect model was applied for meta-analysis. We used the mean difference (MD) and odds ratio (OR) to compare continuous and dichotomous variables, respectively. Funnel plots were used to evaluate the publication bias. The confidence interval (CI) was set at 95% and the *P* value < 0.05 was identified as statistically significant.

## Results

### Eligible studies and characteristics

**Fig. 1:**
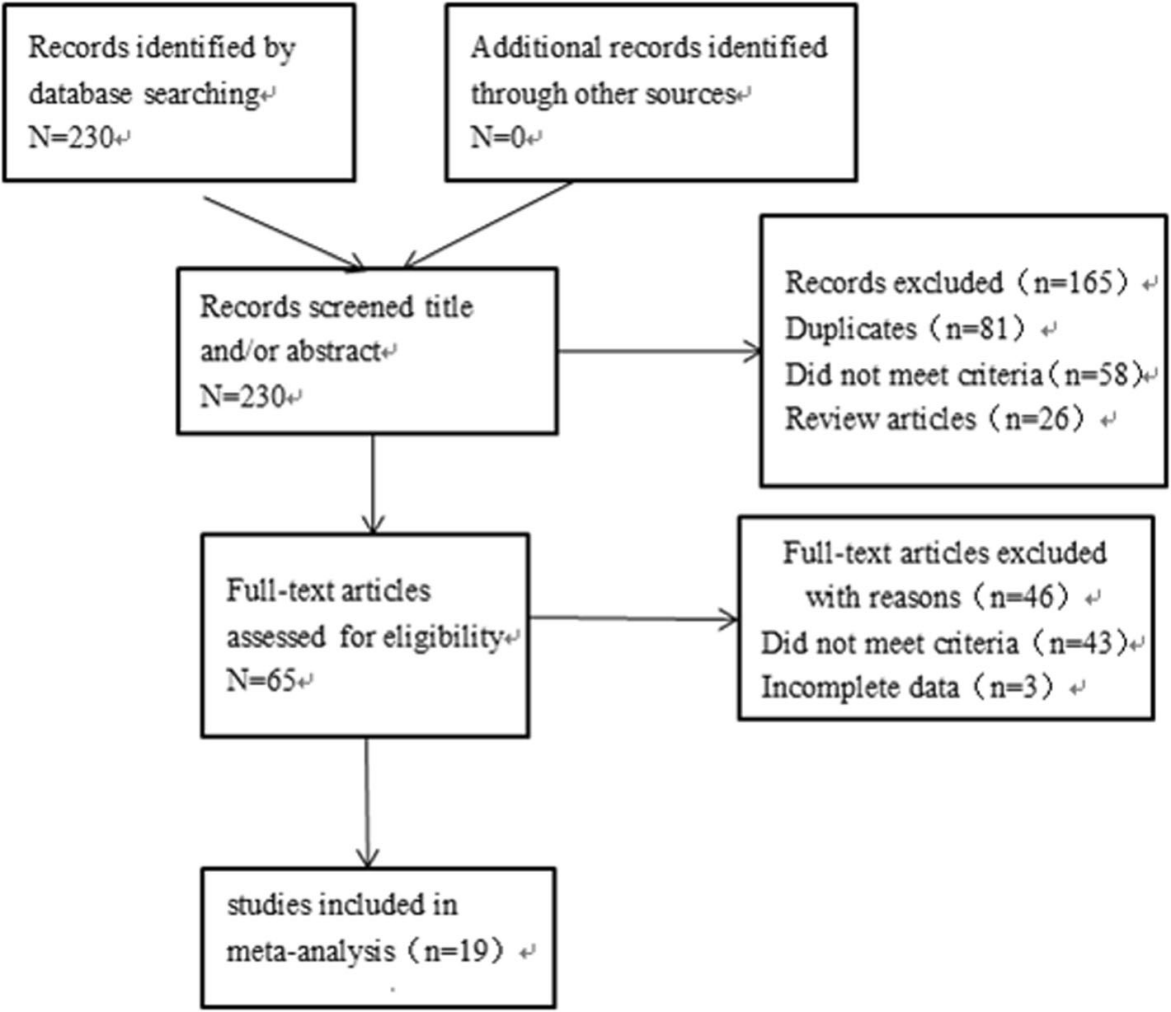
Flow diagram of the study selection process

A total of 19 studies with 2651 patients were included in this meta-analysis, 1369 underwent EBRT and 1282 underwent CTURBT. The characteristics of the included articles were presented in Table 1. Of all articles, four were RCTs [7, 12–14], three were prospective studies [11, 15, 19], and 12 were retrospective studies [6, 8–10, 16–18, 20–24]. Laser, “button” shape electrode, loop electrode, or HybridKnife were used in the EBRT group. Loop electrode was used in CTURBT group. Figure 1 summarized the inclusion process. We performed intravesical chemotherapy or Bacille Calmette-Guerin (BCG) for postoperative patients, mitomycin was used in five studies [8, 9, 15–17], epirubicin was used in seven studies [6, 7, 10, 12, 14, 23, 24], pirarubicin was used in five studies [13, 18, 20–22], BCG was used in one study [11], and BCG combined epirubicin [19] was used in one study.

**Table 1:** Characteristics of included studies

Study	Study design	LOE	Surgical method	Tumor size		No. of patients	Grade			T-stage			P/M/E/BCG	NOS score
				EBRT	CTURBT	EBRT CTURBT		EBRT	CTURBT		EBRT	CTURBT		
Zhu et al. 2008	Retrospective	3b	Holmium laser/CTURBT	≤ 3 cm 95 > 3 cm 6	104 7	101/111	1 2 3	36 54 9	38 63 10	Ta T1	67 34	70 41	M	*******
Zhong1 et al. 2010	Retrospective	3b	2-micron laser/CTURBT	2.23	1.54	30/42	LMP LG HG	4 21 5	7 26 9	Ta T1 Cis	23 5 2	30 8 4	E	*******
Zhong2 et al. 2010	Retrospective	3b	Holmium laser/CTURBT	1.38	1.54	25/42	LMP LG HG	3 18 4	7 26 9	Ta T1 Cis	19 5 1	30 8 4	E	*******
Liu et al. 2013	RCT	2b	2-micron laser/MCTURBT	1.31	1.28	64/56	LMP LG HG	11 46 7	10 41 5	Ta T1	37 27	34 22	E	–
Sureka et al. 2015	Prospective	2b	EBRT/MCTURBT	2.8	3.3	21/24	NM	NM	NM	Ta T1	12 9	13 11	BCG	*******
Chen et al. 2015	RCT	2b	2-micron laser/CTURBT	2.6	2.3	71/71	LMP LG HG	5 43 23	9 45 17	Ta T1 Cis	43 25 3	55 15 1	E	–
Xu et al. 2015	RCT	2b	2-micron laser/MCTURBT	≤ 3 cm 81 > 3 cm 18	≤ 3 cm 79 > 3 cm 15	116/113	1 2 3	50 39 10	48 41 5	Ta T1	91 8	82 12	P	–
Zhang et al. 2015	RCT	2b	2-micron laser/BCTURBT	≤ 3 cm 98 > 5 cm 51	≤ 3 cm 95 > 5 cm 48	149/143	G0 G1 G2	87 54 8	75 60 8	Ta T1	10 6 43	107 36	E	–
Cheng et al. 2017	Retrospective	3b	KTP laser/CTURBT	1.65	1.5	34/30	Low High	19 2	20 0	Ta T1	14 16	13 15	M	*******
Zhang et al. 2017	Retrospective	3b	EBRT/MCTURBT	≤ 3 cm 32 > 3 cm 8	≤ 3 cm 38 > 3 cm 12	40/50	LMP LG HG	9 22 9	12 23 15	Ta T1	15 25	27 23	P	******
Balan et al. 2018	Prospective	2b	EBRT/MCTURBT	1.82	1.69	45/45	NM	NM	NM	Ta T1	24 21	23 22	E and BCG	******
Li et al. 2018	Retrospective	3b	TH laser/CTURBT	2.39	2.15	136/120	NM	NM	NM	NM	NM	NM	P	*******
Yang et al. 2013	Retrospective	3b	KTP laser/CTURBT	≤ 3 cm 24 > 3 cm 4	≤ 3 cm 26 > 3 cm 6	28/32	1 2 3	15 10 3	16 10 6	Ta T1	8 20	7 25	E	******
Tao et al. 2013	Retrospective	3b	KTP laser/CTURBT	2.1	1.9	74/84	LMP LG HG	9 60 5	10 68 6	Ta T1 Cis	50 23 1	61 21 2	E	******
Cheng et al. 2018	Retrospective	3b	HybridKnife/CTURBT	2.5	2.8	95/98	LMP LG HG	5 48 40	8 37 48	Ta T1	52 43	54 44	P	*******
D’souza et al. 2016	Retrospective	3b	Holmium laser/MCTURBT	1.41	1.58	27/23	LMP LG HG	5 20 2	4 16 3	Ta T1	16 11	15 8	M	*******
Song et al. 2010	Retrospective	3b	Holmium laser/MCTURBT	1.85	1.74	64/51	LMP LG HG	5 39 20	4 33 14	Ta T1 Cis	36 23 5	30 17 4	M	*******
Huang1 et al. 2016	Retrospective	3b	2-micron laser/MCTURBT	1.63	1.53	70/70	LMP LG HG	20 40 10	18 46 6	Ta T1 Cis	40 23 7	35 27 8	E	********
Huang2 et al. 2016	Retrospective	3b	Holmium laser/MCTURBT	1.58	1.53	70/70	LMP LG HG	15 48 7	18 46 6	Ta T1 Cis	37 28 5	35 27 8	E	********
Xu et al. 2017	Retrospective	3b	Holmium laser/CTURBT	2.3	2.2	26/44	LMP LG HG	4 14 8	3 28 13	Ta T1	10 12	25 16	P	*******
Chen et al. 2016	Prospective	2b	Green laser/CTURBT	1.85	1.71	83/75	LMP LG HG	12 61 10	8 55 12	Ta T1	70 13	64 11	M	*******

*RCT* randomized control trial, *NM* not mention, *CTURBT* conventional transurethral resection of bladder tumor, *MCTURBT* monopolar conventional transurethral resection of bladder tumor, *BCTURBT* bipolar conventional transurethral resection of bladder tumor, *EBRT* en bloc resection of bladder tumor, *LMP* low malignant potential, *LG* low grade, *HG* high grade, *P* pirarubicin, *M* mitomycin, *E* epirubicin, *BCG* Bacille Calmette-Guerin

### Quality assessment of included studies

**Fig. 2:**
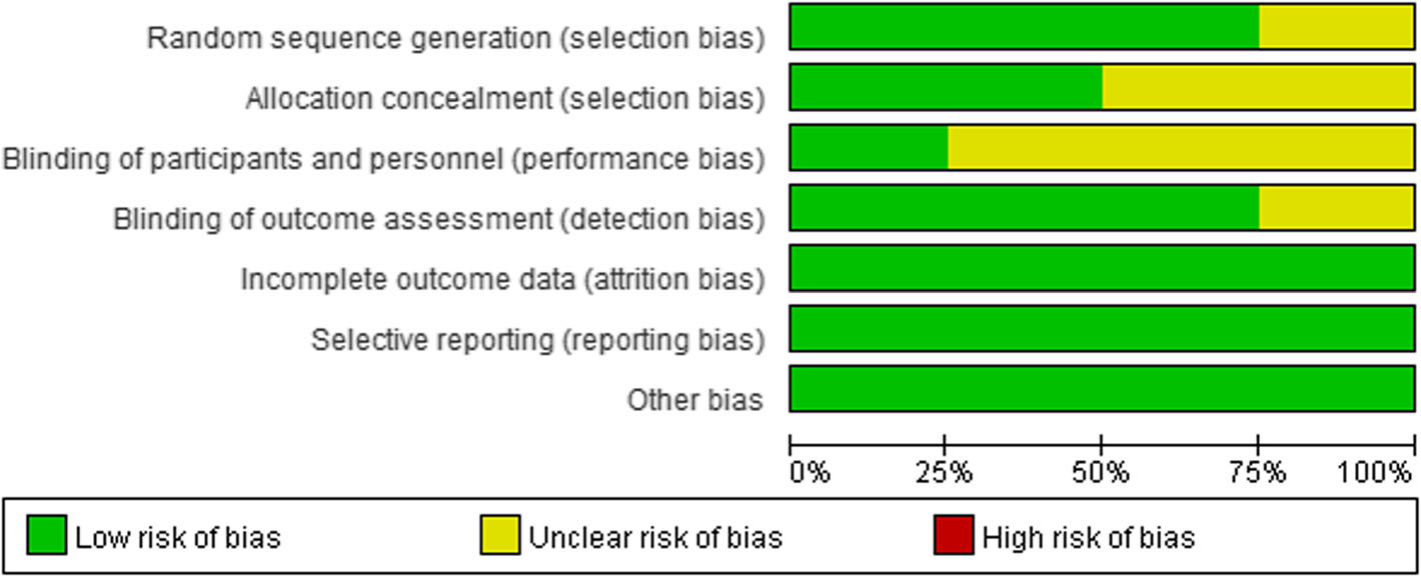
Overall quality assessment for included randomized controlled trials

We described the level of evidence of the 19 articles in Table 1. According to the risk of bias graph, four RCTs were all considered high-quality studies, with > 3 kinds of bias were at low risk (Fig. 2). Furthermore, 11 non-randomized studies [6, 8, 9, 11, 15–17, 20–22, 24] were considered of high quality due to the score ≥ 7 stars (Table 1).

### Operative time, hospitalization time, catheterization time

Twelve articles reported the HT, 15 articles reported the AT, and 17 articles reported the OT. Based on 12 included articles [6–9, 12, 13, 15, 16, 18, 21, 22, 24], the patients treated with EBRT had significantly decreased HT compared with CTURBT (*P* < 0.00001). The MD was − 1.30, in favor of EBRT [(random effect) 95% confidence interval [Cl], − 1.70 to − 0.91; *P* < 0.00001] (Table 2). Similarly, 15 articles [6–10, 12, 13, 15, 16, 18, 19, 21–24] provided evidence suggesting that the difference in AT was significant between the two groups [MD, − 0.97; 95% [Cl], − 1.30 to − 0.64; *P* < 0.00001] (Table 2). Furthermore, no significant difference was found in terms of OT [MD, − 0.56; 95% [Cl], − 2.16 to − 1.04; *P* = 0.49] [6–10, 12–16, 18, 21–23] (Table 2). While there was a high heterogeneity in all comparisons, which may come from the patient demographics, difference in types of surgery, and tumor characteristics.

**Table 2:** The main result of this meta-analysis

Endpoint	No. of studies	Heterogeneity		OR/MD[95%CI]
		*I*^2^ %	p	
Operation time	17	72	0.49	− 0.56 [− 2.16, 1.04]
Catheterization time	17	95	< 0.00001	− 0.97 [− 1.30, − 0.64]
Hospitalization time	14	95	< 0.00001	− 1.30 [− 1.70, − 0.91]
Obturator nerve reflex	16	43	< 0.00001	0.12 [0.07, 0.19]
Bladder perforation	16	0	< 0.00001	0.17 [0.09, 0.35]
Bladder irritation	5	41	< 0.00001	0.21 [0.14, 0.32]
Postoperative complications	11	0	0.01	0.40 [0.20, 0.82]
Residual tumor on the base	2	0	0.44	0.47 [0.07, 3.27]
Bladder detrusor muscle	4	85	0.16	3.59 [0.60, 21.63]

### Complications

Where reported, the main intraoperative and postoperative side effects included obturator nerve reflex [7–10, 12, 13, 15, 17–24], bladder perforation [6–10, 13, 15, 16, 18, 20–24], bladder irritation [6, 7, 9, 16], urethral stricture, bleeding, and fever [6, 7, 9, 14–16, 20, 21, 24]. The characteristic of tumors at the lateral walls in each article are similar. According to the pooled articles, the patients treated with EBRT had significantly decreased the obturator nerve reflex[OR, 0.12; 95% [Cl], 0.07 to 0.19; *P* < 0.00001] (Table 2), bladder perforation[OR, 0.17; 95% [Cl], 0.09 to 0.35; *P* < 0.00001] (Table 2), bladder irritation [OR, 0.21; 95% [Cl], 0.14 to 0.32; *P* < 0.00001] (Table 2), and postoperative complications [OR, 0.40; 95% [Cl], 0.20 to 0.82; *P* = 0.01] (Table 2) when compared with CTURBT. There was no significant heterogeneity among the all comparisons.

### The residual tumor on the base

The repeated biopsy on the base was performed by Zhang et al. [14, 18] after the tumor was resected. Two pooled articles [14, 18] showed that CTURBT had similar residual tumor rate compared with EBRT [OR, 0.47; 95% [Cl], 0.07 to 3.27; *P* = 0.44] (Table 2).

### Bladder detrusor muscle

The bladder detrusor muscle in the specimen was showed in four articles [14, 18, 20, 21]. Although there were 94% and 86.9% positive rate in EBRT and CTURBT group, respectively, no significant difference was found between two groups [OR, 3.59; 95% [Cl], 0.6 to 21.63; *P* = 0.16] (Table 2).

### Twelve-month recurrence

**Fig. 3:**
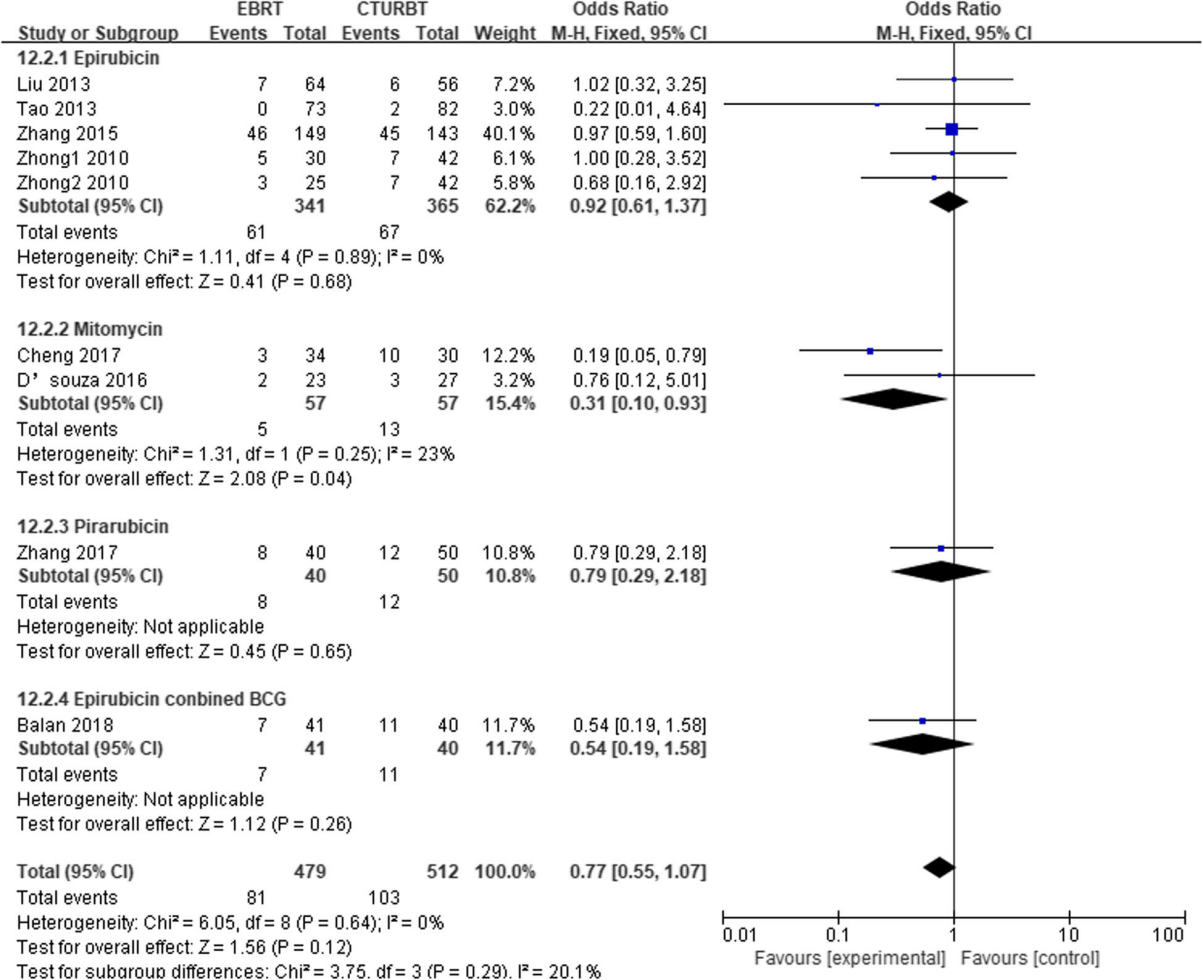
Twelve-month recurrence based on the different therapy of intravesical instillations

The 12-month recurrence rate between groups was compared in eight studies [6, 7, 10, 14, 16, 18–20]. Two groups had a similar 12-month recurrence rate [OR, 0.77; 95% [Cl], 0.55 to 1.07; *P* = 0.12]. In subgroup analyses (Fig. 3) according to the different therapy of intravesical instillations (Table 3), no difference was found between groups using epirubicin [OR, 0.92; 95% [Cl], 0.61 to 1.37; *P* = 0.68], pirarubicin [OR, 0.79; 95% [Cl], 0.29 to 2.18; *P* = 0.65], or epirubicin combined with BCG [OR, 0.54; 95% [Cl], 0.19 to 1.58; *P* = 0.26] for NMIBC. However, based on two articles using mitomycin, the pooled OR is 0.31 [OR (fixed effect) 95% Cl, 0.10 to 0.93; *P* = 0.04], which indicated that EBRT had a lower 12-month recurrence rate than CTURBT. Subgroup was also performed based on the study type. Table 3 summarized the results.

**Table 3:** RCT, randomized controlled trail; BCG, Bacille Calmette-Guerin; NA, not applicable

	Subgroup	No. of studies	Heterogeneity		OR/MD [95%CI]
			*I*^2^ %	*p*	
12-month recurrence	RCT	2	0	0.93	0.98 [0.62, 1.55]
	Prospective	1	NA	0.26	0.54 [0.19, 1.58]
	Retrospective	6	0	0.06	0.59 [0.33, 1.03]
	Epirubicin	5	0	0.68	0.92 [0.61, 1.37]
	Mitomycin	2	23	0.04	0.31 [0.10, 0.93]
	Pirarucin	1	NA	0.65	0.79 [0.29, 2.18]
	Epirubicin + BCG	1	NA	0.26	0.77 [0.55, 1.07]
24-month recurrence	RCT	2	0	0.19	0.70 [0.41, 1.19]
	Prospective	2	19	0.02	0.43 [0.21, 0.89]
	Retrospective	10	0	0.006	0.64 [0.47, 0.88]
	Epirubicin	7	0	0.13	0.71 [0.45, 1.10]
	Mitomycin	4	0	0.01	0.61 [0.41, 0.90]
	Pirarucin	2	0	0.14	0.65 [0.36, 1.16]
	BCG	1	NA	0.03	0.24 [0.07, 0.84]
36-month recurrence		4	58	0.32	0.72 [0.37, 1.39]
Same site recurrence		5	0	0.10	0.49 [0.21, 1.14]
Recurrence	Low-risk	5	0	0.96	1.01 [0.63, 1.63]
	Intermediate-risk	4	0	0.26	0.76 [0.47, 1.23]
	High-risk	3	0	0.76	0.82 [0.24, 2.85]

### Twenty-four-month recurrence

**Fig. 4:**
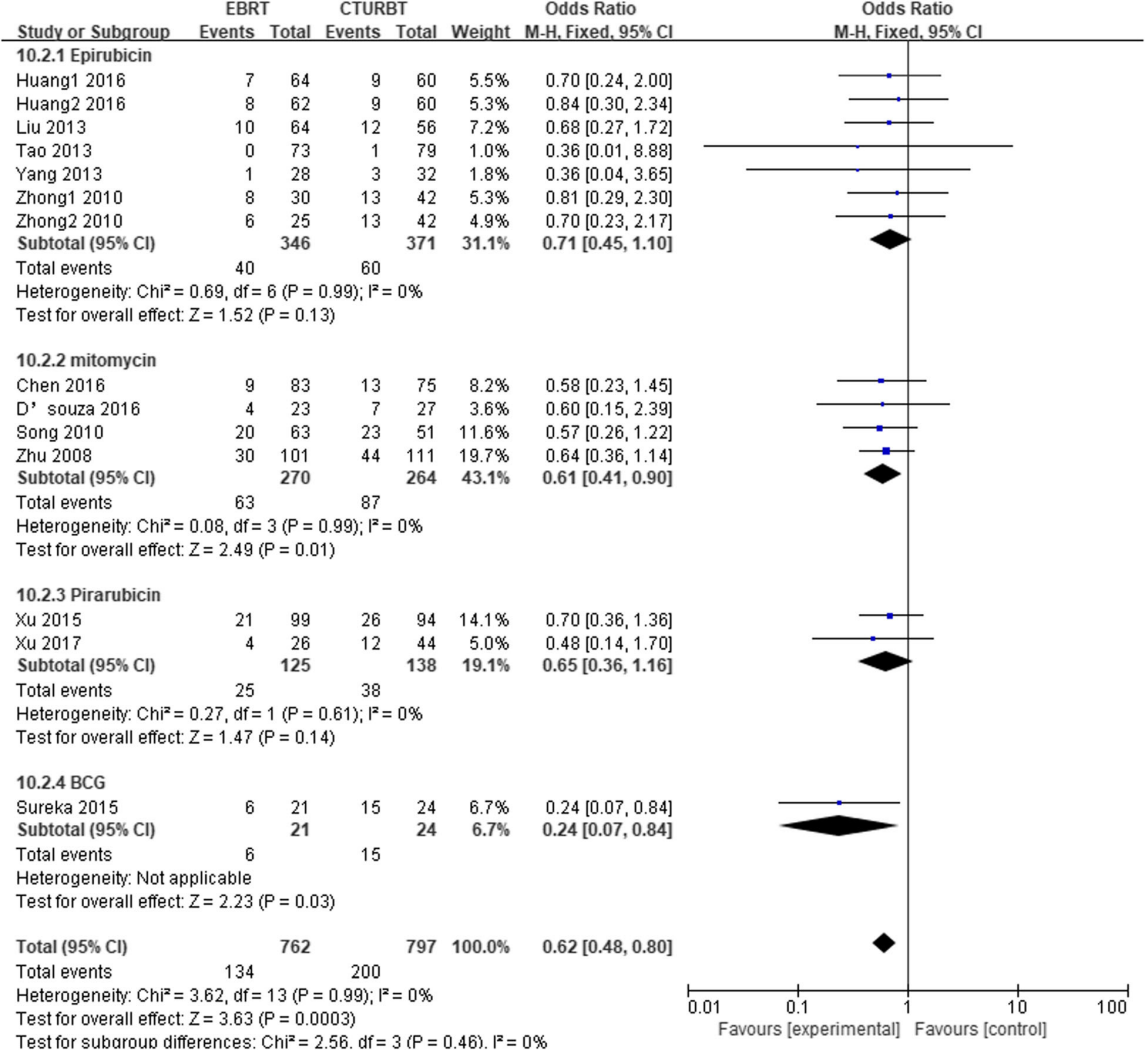
Twenty-four-month recurrence rate based on the different therapy of intravesical instillations

A total of 1559 patients were included, 762 underwent EBRT and 797 underwent CTURBT. In meta-analysis, 12 pooled studies [6–11, 13, 15, 16, 22–24] showed that CTURBT apparently had a higher 24-month recurrence compared with EBRT [OR, 0.62; 95% [Cl], 0.48 to 0.80; *P* = 0.0003]. Subgroup analyses were conducted based on the different therapy of intravesical instillations. There were no significantly difference between two groups in the epirubicin subgroup [OR, 0.71; 95% [Cl], 0.45 to 1.10; *P* = 0.13] or pirarubicin subgroup [OR, 0.65; 95% [Cl], 0.36 to 1.16; *P* = 0.14]. However, in mitomycin [OR, 0.61; 95% [Cl], 0.41 to 0.90; *P* = 0.01] and BCG subgroups [OR, 0.24; 95% [Cl], 0.07 to 0.84; *P* = 0.03] (Fig. 4), the pooled results showed that patients treated with EBRT had a lower 24-month recurrence. No significant heterogeneity was showed in all comparisons. Subgroup was also performed based on the study type. Table 3 summarized the results.

### Thirty-six-month recurrence

**Fig. 5:**
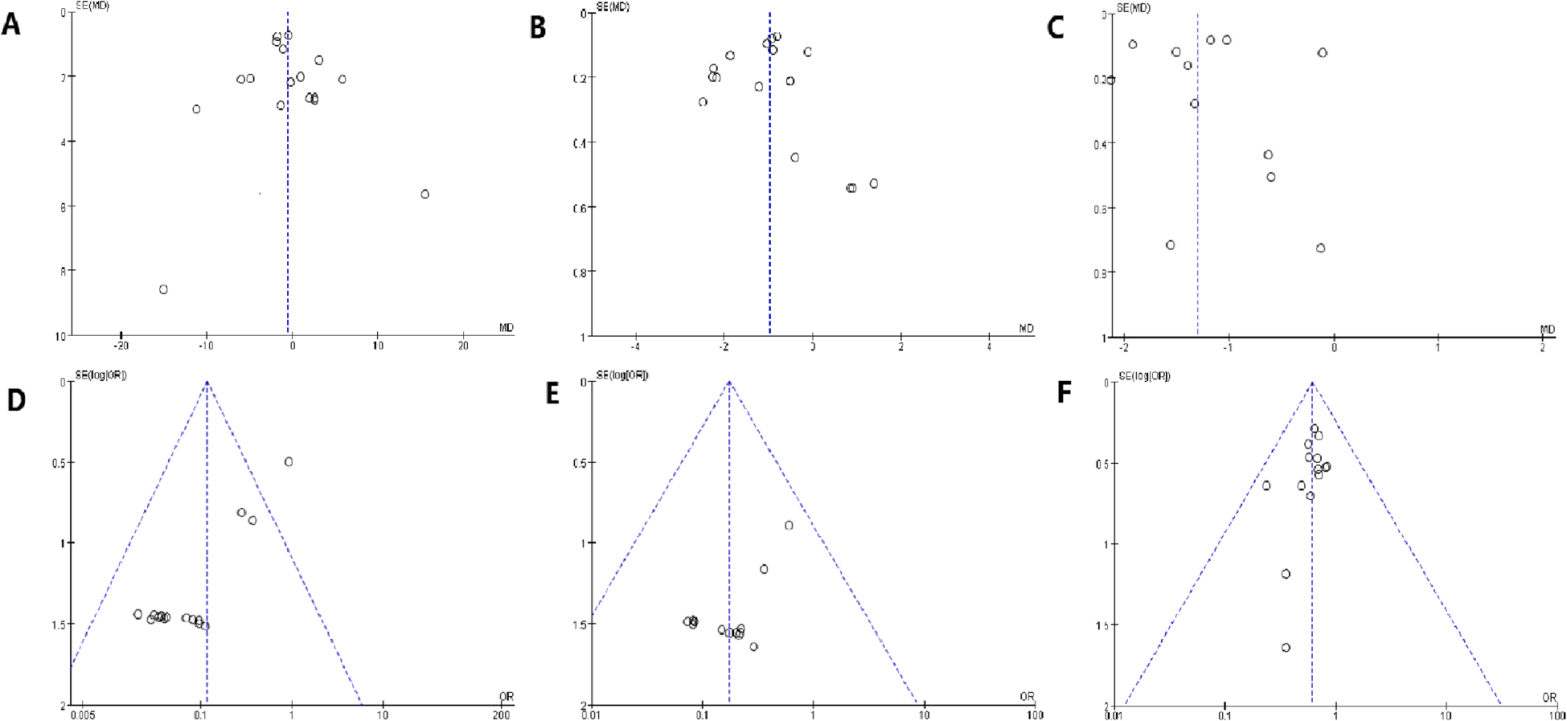
Funnel plot. **a** Operation time. **b** Catheterization time. **c** Hospitalization time. **d** Obturator nerve reflex. **e** Bladder perforation. **f** 24-month recurrence rate

Four articles were analyzed for 36-month recurrence as the outcome. Four pooled studies [7, 14, 16, 20] including 203 patients showed that there were 29.6% and 32.4% 36-month recurrence rate in EBRT and CTURBT group, respectively, but no significant difference was found between two groups [OR, 0.72; 95% [Cl], 0.37 to 1.39; *P* = 0.32] (Table 3). Our pooled estimate showed significant heterogeneity (*I*^2^ = 58%), which may come from the Cheng et al. study. Because only HybridKnife was used for EBRT by Cheng et al., laser was used by the others (Fig. 5).

### Same site recurrence

Five included articles [11, 13, 18, 19, 23] evaluated the same site recurrence rate with the follow-up time between 12 and 38 months. Compared with the CTURBT, EBRT had a lower same site recurrence rate, while no significant difference was found between two groups [OR, 0.49; 95% [Cl], 0.21 to 1.14; *P* = 0.10] (Table 3).

### Low-risk, intermediate-risk, high-risk

According to the EAU guidelines [3], patients were divided into the low-risk group [6, 9, 13, 14], intermediated-risk [6, 9, 13, 14], and high-risk group [9, 13, 14]. The main evidence for grouping was tumor size, tumor numbers, tumor category, and tumor grade. No significant difference was found in terms of recurrence rate between 12 and 36 months in the low-risk group [OR, 1.01; 95% [Cl], 0.63 to 1.63; *P* = 0.96], intermediated-risk group [OR, 0.76; 95% [Cl], 0.47 to 1.23; *P* = 0.26], and high-risk group [OR, 0.82; 95% [Cl], 0.24 to 2.85; *P* = 0.76] (Table 3).

### Publication bias

According to the funnel plots, although a publication bias exists in obturator nerve reflex. No significant publication bias was detected for our other results.

## Discussion

This meta-analysis showed that compared with CTURBT, EBRT for NMIBC had a significantly lower AT, HT, obturator nerve reflex, bladder perforation, bladder irritation, postoperative complications, and 24-month recurrence. While no significant difference was found in terms of OT, the ratio of bladder detrusor muscle found in the specimen, the residual tumor on the base, 12-month recurrence rate, 36-month recurrence rate, and the ratio of the same site recurrence. In the mitomycin subgroup, EBRT was significantly superior to CTURBT in terms of 12/24-month recurrence rate. Similarly, in the prospective subgroup and retrospective subgroup, EBRT had a lower 24-month recurrence rate than CTURBT. However, no significant difference was found in the low, intermediate, and high-risk group in the light of 12–36-month recurrence rate. Therefore, the pooled data lead support to EBRT as a superior method for NMIBC.

As technology progresses, CTURBT is widely used in the treatment of NMIBC. However, there remain some limitations needing to be overcome. Firstly, it is inevitable for a tumor with a diameter of over 3 cm to be resected piece-by-piece and then the fragments would be washed out through the cystoscope sheath naturally, which is contradictory to the tumor-free principle. Secondly, detrusor muscle is one of the criteria to assess the completeness of resection. The eschar in the specimen caused by electric coagulation would affect the accuracy of tumor infiltration for its depth, grading, and staging. Thirdly, there is a real possibility for such complications as obturator nerve reflex and bladder perforation to occur during the resection of lateral wall tumors [29, 30]. EBRT is a modified method for NMIBC. According to EAU guidelines, it is capable of providing high-quality specimen including muscle layer in 96–100% of the existing cases [31–33]. Meanwhile, EBRT could help reduce various complications, for example, obturator nerve reflex, bladder perforation, bladder irritation, and urethral stricture. Despite no significant difference of bladder detrusor muscle present in the specimen observed in our meta-analysis, the residual tumor on the base and same site recurrence rate between groups, detrusor muscle positive rate in EBRT were found superior to CTURBT group (94% vs. 86.9%). Similarly, EBRT revealed a lower residual tumor on the base (0.53% vs. 1.55%) and same site recurrence rate (3.74% vs. 8.69%).

EBRT showed a shorter HT, AT, fewer complications, and lower 24-month recurrence rate than CTURBT in the treatment of NMIBC, which is a similar conclusion to that drawn in another meta-analysis published in 2016 [25]. In our meta-analysis, moreover, attempt was made to explore the differences between the two groups with regard to the ratio of bladder detrusor muscle found in the specimen, the residual tumor on the base, 12-month recurrence rate, 36-month recurrence rate, and the ratio of the same site recurrence. Furthermore, subgroups were set up based on the types of study and the characteristics of tumor. Despite the expansion of sample size and research area, the validity of our results was limited by the 12 retrospective studies.

Intravesical chemotherapy or Bacille Calmette-Guerin(BCG)was performed for postoperative patients. The duration and dosage of postoperative therapy varied. Therefore, based on the different therapies of intravesical instillations, a subgroup analysis was conducted to determine the 12/24-month recurrence rate. As revealed by the pooled studies, with regard to 12/24-month recurrence rate, EBRT was clearly superior to CTURBT for the patients receiving mitomycin. In other subgroups, EBRT showed a lower recurrence rate, despite no statistical significance, which suggested that postoperative adjuvant therapy is a crucial influencing factor for the prognosis.

Based on the study type, a subgroup analysis was carried out to evaluate the 12/24-month recurrence rate. Despite no significant difference found in respect of 12-month recurrence rate, EBRT exhibited a lower 24-month recurrence rate than CTURBT in the prospective subgroup (*P* = 0.02) and retrospective subgroup (*P* = 0.006), which is statistically significant. Furthermore, in the RCTs subgroup, two pooled studies revealed that 19% and 25% 24-month recurrence rate were observed in EBRT and CTURBT group, which indicates the advantages of EBRT.

The heterogeneity of each study on the pooled results was evaluated by excluding single study sequentially, which led to the results suggesting that the heterogeneity remained at a high level in respect of OT, AT, HT, and bladder detrusor muscle, which is speculated to result from the differences in the characteristics of tumor, demographics, and surgical technology. However, as for 36-month recurrence, the heterogeneity declined from 58 to 0 when the study performed by Cheng et al. was excluded, which indicates that this study should be responsible for the heterogeneity of our included studies. Reading the articles, the EBRT group with the application of HybridKnife had a significantly lower 36-recurrence rate than CTURBT (*P* = 0.008), while no difference was found in other studies when laser was applied. This might account for this situation. Therefore, the research conducted by Cheng et al. was excluded. According to the results of the sensitivity analysis, there was no significant difference observed as before [OR, 1.02; 95% [Cl], 0.70 to 1.49; *P* = 0.91].

However, it is worth mentioning some limitations on this meta-analysis. Firstly, this meta-analysis involves a combination of prospective and retrospective studies, which has a potential to result in a significant bias across the studies. Secondly, the characteristics of tumor in our included articles show difference. Some articles included Ta and T1, while other articles involved Ta, T1, and Tis. Moreover, some patients had multiple tumors (including all patients in the study by Liu et al.) and there was a lack of information on how many of them were resected en bloc. Thirdly, the mean follow-up time was as little as 12–36 months. As demonstrated by the pooled studies, EBRT showed a lower 24-month recurrence rate compared with those treated with CTURBT. However, there was no significant difference observed in respect of 12- or 36-month recurrence rate. A sufficiently long follow-up time should be allowed to better compare the recurrence-free survival among different groups. Fourthly, the articles included in this study were restricted to those published in Embase, PubMed, and Cochrane Central Register, as a result of which case-reports, reviews, abstracts, animal experiments and letters were excluded, which is possible to cause potential selection bias and language bias. Fifthly, not all pathology departments put in place a routine to report whether there is muscle present or not and only four out of the 19 studies reported it. Furthermore, more studies should be performed to compare the safety and efficacy of EBRT against CTURBT based on the classification into low-risk, intermediate-risk, and high-risk. Finally, it is speculated that tumor recurrence rate could be affected by other influencing factors such as surgeon, available equipment, surgical team, smoking, and gene. All the limitations as mentioned above could compromise the value of our meta-analysis.

## Conclusion

Our pooled studies showed that EBRT had a significantly lower AT, HT, intraoperative and postoperative complications, and 24-month recurrence rate than those treated with CTURBT, but due to the lack of randomization and selection bias, randomized studies will need to be performed to confirm our findings. Although, EBRT trended toward having a higher ratio of bladder detrusor muscle in the specimen, a lower ratio of residual tumor on the base and the same site recurrence, a lower ratio of 12-month and 36-month recurrence than CTURBT, but the differences did not reach statistical significance.

## Data Availability

Not applicable.

## Abbreviations

AT: Catheterization time
BCG: Bacille Calmette-Guerin
BCTURBT: Bipolar conventional transurethral resection of bladder tumor
CI: Confidence interval
CTURBT: Conventional transurethral resection of bladder tumor
EBRT: En bloc transurethral resection of bladder tumor
HT: Hospitalization time
MCTURBT: Monopolar conventional transurethral resection of bladder tumor
MD: Mean difference
NMIBC: Nonmuscle-invasive bladder cancer
OR: Odds ratio
OT: Operation time
RCT: Randomized controlled trial
